# Complete mitochondrial genome of the hydrothermal vent shrimp *Rimicaris variabilis* (Decapoda: Caridea: Alvinocarididae) from the North Fiji basin

**DOI:** 10.1080/23802359.2019.1674717

**Published:** 2019-10-11

**Authors:** Won-Kyung Lee, Se-Joo Kim, Se-Jong Ju, Bo Kyeng Hou

**Affiliations:** a Genome Editing Research Center, Korea Research Institute Bioscience and Biotechnology, Daejeon, Korea;; b Global Ocean Research Center, Korea Institute of Ocean Science and Technology, Busan, Korea;; c Marine Biology Major, University of Science & Technology, Daejeon, Korea

**Keywords:** *Rimicaris variabilis*, Alvinocarididae, hydrothermal vent shrimp, mitochondrial genome, North Fiji Basin

## Abstract

The family Alvinocarididae is the monophyletic taxon which lives restrictively at chemosynthesis-based environments in the deep-sea. Here, for the first time, we report the complete mitogenome of the alvinocaridid vent shrimp *Rimicaris variabilis* from the North Fiji Basin. The mitogenome was 15,909 bp in length, with 65.6% AT content. Its protein-coding gene organization was typical of other alvinocaridid shrimps. Based on the phylogenetic tree, *R. variabilis* was most closely related to *Shinkaicaris leurokolos*, rather than with other *Rimicaris* species. To resolve this incongruence between traditional morphological classification and molecular analyses, further mitogenomic analysis of undetermined alvinocaridid taxa is necessary.

The family Alvinocarididae is restricted to the chemosynthesis-based environments (hydrothermal vents and cold seeps) and composed of 32 species belonging to eight genera (Martin and Haney 2005; Vereshchaka et al. 2015). Although the family is confirmed as a monophyletic taxon in caridean shrimps based on molecular phylogenetic studies, the phylogenetic relationship among its members is not completely resolved yet (Vereshchaka et al. 2015; Sun et al. 2018).

The alvinocaridid shrimp *Rimicaris* is characterized by reduced and dorsoventrally flattened rostrum, and lack of postrostral carina on the carapace (Komai and Giguère 2019). The genus is composed of 10 species including six *Chorocaris* species synonymised by Vereshchaka et al. (2015). As of 5 September 2019, mitochondrial genome (mitogenome) of two *Rimicaris* species, *R. exoculata* and *R. kairei*, are registered in GenBank database. To elucidate the phylogenetic relationship among alvinocaridid shrimps, we determined the mitogenome of *R. variabilis*.

*Rimicaris variabilis* specimen was collected from hydrothermal vent in the North Fiji Basin (18°49′S and 173°30′E; 2,722 m depth) in November 2016 (Figure 1(A)). Genomic DNA extraction, sequencing, gene annotation, and phylogenetic analyses followed the methods of Kim et al. (2017). The specimen used for the analysis of mitogenome is deposited at the National Marine Biodiversity Institute of Korea (accession no. MABIK CR00246489).

**Figure 1.**
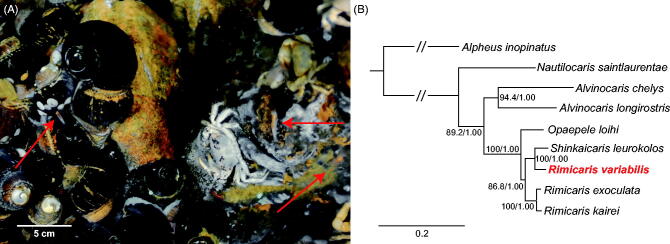
(A) A photograph of the hydrothermal vent chimney in the North Fiji Basin of the Southwestern Pacific Ocean where *R. variabilis* was collected. Red arrows indicate *R. variabilis*. (B) Phylogenetic relationship of *Rimicaris variabilis* with other alvinocaridid shrimps and an outgroup (*A. inopinatus*) based on 13 mitochondrial protein-coding genes using maximum-likelihood (ML) and Bayesian inference (BI) methods. The model GTR + I + G was selected as the best evolutionary model using jModelTest 2.1.4. Numbers on internodes indicate maximum likelihood bootstrap proportions and Bayesian posterior probabilities, respectively. The accession numbers of shrimps are as follows: *Alpheus inopinatus*, NC_041151; *Nautilocaris saintlaurentae*, NC_021971; *Alvinocaris chelys*, NC_018778; *Alvinocaris longirostris*, NC_020313; *Opaepele loihi*, NC_020311; *Shinkaicaris leurokolos*, NC_037487; *Rimicaris variabilis*, MN419306; *Rimicaris exoculata*, NC_027116; *Rimicaris kairei*, NC_020310.

The complete mitogenome of *R. variabilis* was 15,909 bp in length (accession no. MN419306; 65.6% AT content) similar to its relatives, *R. exoculata* (15,902 bp; 65.7% AT content) and *R. kairei* (15,900 bp; 53.8% AT content). It contained 13 protein-coding genes (PCGs), two ribosomal RNAs (rRNAs), 22 transfer RNAs (tRNAs), and a putative control region. The gene organization was identical to that of other alvinocaridid shrimps.

All PCGs had an ATN start codon except for ND5 (GTG). Also, most of them terminated with a complete stop codon (TAA or TAG), while COX1, COX2, ND4, and ND5 had an incomplete stop codon (T––). The 16S and 12S rRNAs were 1,311 bp (70.0% AT content) and 866 bp (71.5% AT content) in length, respectively. The size of tRNA genes varied between 63 and 72 bp. A putative control region (1,008 bp; 83.0% AT content) was located between the 12S rRNA and tRNA*^Ile^*.

Phylogenetic trees were constructed with 13 PCGs of eight shrimps, one alpheid as an outgroup and seven alvinocaridids, using maximum likelihood (ML) and Bayesian inference (BI) (Figure 1(B)) methods. ML and BI tree topologies were identical, and they were similar to those from previous studies that used six genetic markers (Sun et al. 2018). Based on the tree, the monophyly of *Opaepele*, *Shinkaicaris*, and *Rimicaris* was strongly supported by the high supporting values (100% bootstrap proportion (BP) and 1.00 Bayesian posterior probability (BPP)). However, three *Rimicaris* species were not monophyletic. Instead, *R. variabilis* was most closely related to *S. leurokolos* (100% BP and 1.00 BPP; 95.6% identity for whole mitogenome).

To resolve this incongruence between traditional morphological classification and molecular phylogenetics in Alvinocarididae, further mitogenomic analysis of undetermined taxa is required.
